# Draft Genome Sequence of the Fungus *Penicillium brasilianum* (Strain LaBioMMi 136), a Plant Endophyte from *Melia azedarach*

**DOI:** 10.1128/MRA.01235-18

**Published:** 2018-11-29

**Authors:** Taícia Pacheco Fill, Jéssica Fernanda Baretta, Luiz Gonzaga Paula de Almeida, Iran Malavazi, Louise Teixeira Cerdeira, Markiyan Samborskyy, Ana Tereza Ribeiro de Vasconcelos, Peter Leadlay, Edson Rodrigues-Filho

**Affiliations:** a Instituto de Química, Universidade Estadual de Campinas, Campinas, São Paulo, Brazil; b Departamento de Química, Universidade Federal de São Carlos, São Carlos, São Paulo, Brazil; c Departamento de Genética e Evolução, Universidade Federal de São Carlos, São Carlos, São Paulo, Brazil; d Department of Biochemistry, University of Cambridge, Cambridge, United Kingdom; e Department of Clinical Analysis, School of Pharmacy, University of São Paulo, São Paulo, São Paulo, Brazil; f Laboratório Nacional de Computação Científica, Petrópolis, Rio de Janeiro, Brazil; Vanderbilt University

## Abstract

Penicillium brasilianum (strain LaBioMMi 136) has been reported to be a great producer of secondary metabolites and a source of enzymes of biotechnological interest. Here, we report the draft genome sequence of P. brasilianum (strain LaBioMMi 136), isolated as an endophyte from the plant Melia azedarach (family Meliaceae).

## ANNOUNCEMENT

Penicillium brasilianum (class Eurotiomycetes, phylum Ascomycota), a fungus isolated from different sources, such as soil (1) and plants (2), including onion (3), has been demonstrated to be an important producer of bioactive secondary metabolites, mainly brasiliamides (1, 4, 5), austin-related insecticidal meroterpenes (2, 6, 7), verruculogen-like tremorgenic alkaloids (8), penicillic acid, and spirohexalines, which are novel inhibitors of bacterial undecaprenyl pyrophosphate synthase (9). Its enzymatic potential has also been under investigation. The ability of the fungus to express Baeyer-Villiger monooxygenases was described by Fill et al. (10), while Panagiotou et al. described the production of feruloyl esterase, xylanase, and arabinofuranosidase, which are able to degrade cell wall polymers of selected cereals (11).

The fungus P. brasilianum (strain LaBioMMi 136) was isolated from root bark of Melia azedarach as an endophyte following the procedure described by Santos and Rodrigues-Filho (2). The root bark was separated mechanically from the xylem and washed with water followed by ethanol and then sterilized with 11% aqueous sodium hypochlorite for 1 min. P. brasilianum (strain LaBioMMi 136) was isolated by replication and grew as a bluish-colored culture. For DNA isolation, the fungus was cultivated at a density of 10^7^ conidia ml^−1^ in a chemically defined medium (Czapeck) composed of glucose (30 g liter^−1^), NaNO_3_ (3.0 g liter^−1^), K_2_HPO_4_ (1.0 g liter^−1^), MgSO_4_ · 7H_2_O (0.5 g liter^−1^), KCl (0.5 g liter^−1^), and FeSO_4_ · 7H_2_O (0.01 g liter^−1^). Total genomic DNA was extracted using the DNeasy plant minikit (Qiagen) according to the manufacturer’s instructions.

The P. brasilianum (strain LaBioMMi 136) genome was sequenced by two platforms, 454 (Roche) and MiSeq paired-end 2 × 150 bp (Illumina) sequencing. The Illumina read length was up to 151 bp (paired end). For the first Illumina run (SRA accession number SRR7995492), the total number of reads after adapter and quality trimming was 12,274,914 with an average length of 117 bp. For the second Illumina run (SRA accession number SRR7995491), the total number of reads after adapter and quality trimming was 11,321,146 with an average length of 129 bp. The 454 FLX+ platform produced reads of up to 1,000 bp; the total number of reads was 1,383,158, and the average read length was 581 bp with a total genome coverage of 140×. The 454 coverage was 40×, and the Illumina coverage was 100×. The 454 reads were quality controlled using GSRunBrowser v2.53 (Roche) with default parameters, and for the Illumina reads, MiSeq reporter v2.2 with adapter trimming and FastQC v0.10.1 (default parameters) (http://www.bioinformatics.babraham.ac.uk/projects/fastqc) were used.

The genome was *de novo* assembled using Newbler v2.53 (Roche). The final assembly consists of a genome size of ∼32.9 Mbp covered by 248 scaffolds with an *N*_50_ value of 292,589 bp. The GC content of the genome is 47.75%.

The genome sequence of P. brasilianum (LaBioMMi 136) was automatically annotated using SABIA v2.0 (12) with default parameters and contains 10,352 protein coding sequences with an average length of 1,818 bp, which make up 57.12% of the whole genome.

antiSMASH v3.0 (13) analysis indicated that P. brasilianum (LaBioMMi 136) has the potential to produce a diverse array of natural products. The genome has 42 putative biosynthetic clusters containing, among others, 22 backbone genes, 12 of which are nonribosomal peptide synthetases (NRPSs), 13 polyketide synthases (PKS), 3 terpenes, 2 NRPS/PKS hybrids, 1 hybrid terpene/PKS, and 1 NRPS/terpene, further supporting the great genetic and enzymatic machinery potential for secondary metabolite production.

Our data will contribute to future molecular studies with P. brasilianum, leading to a better understanding of the host-endophyte chemical interactions and its complex secondary metabolite collection.

### Data availability.

This genome sequencing project was uploaded to GenBank and is available under the accession number LJBN00000000. This paper describes the first version of the P. brasilianum genome (LJBN01000000). The first Illumina run is available under the SRA accession number SRR7995492, and the second Illumina run is available under the SRA accession number SRR7995491. The 454 run raw reads are available under SRA accession number SRR7993873.
